# Deep learning-based fully automated detection and segmentation of pelvic lymph nodes on diffusion-weighted images for prostate cancer: a multicenter study

**DOI:** 10.1186/s40644-025-00840-w

**Published:** 2025-03-17

**Authors:** Zhaonan Sun, Pengsheng Wu, Tongtong Zhao, Ge Gao, Huihui Wang, Xiaodong Zhang, Xiaoying Wang

**Affiliations:** 1 Department of Radiology, Peking University First Hospital, No.8 Xishiku Street, Xicheng District, Beijing, 100034 China; 2 Beijing Smart Tree Medical Technology Co. Ltd., Beijing, China

**Keywords:** Prostate cancer, Lymph nodes, Segmentation, Detection, Deep learning

## Abstract

**Background:**

Accurate identification and evaluation of lymph nodes (LNs) in prostate cancer (PCa) patients is crucial for effective staging but can be time-consuming. We utilized a 3D V-Net model to improve the efficiency and accuracy of LN detection and segmentation.

**Methods:**

Utilizing pelvic diffusion-weighted imaging (DWI) scans, the 3D V-Net framework underwent training on a dataset comprising data from a hospital with 1,151 patients, encompassing 32,507 annotated LNs, following data augmentation procedures. Subsequently, external validation was conducted on data from 401 patients across three additional hospitals, encompassing 7,707 LNs. The segmentation performance was evaluated using the Dice similarity coefficient (DSC). The comparison between automated and manual segmentation regarding the short diameter and volume of LNs was conducted using Bland–Altman plots and correlation analysis. The performance for suspicious metastatic LN detection (short diameter > 8 mm) was evaluated using sensitivity, positive predictive value (PPV), and per-patient false-positive rate (FP/vol) at the LN level and sensitivity, specificity, and PPV at the patient level.

**Results:**

In the external validation test dataset, the model achieved a DSC of 0.77–0.82 for all, suspicious, and largest LNs. The model achieved a sensitivity, PPV, and FP/vol of 60.1% (95% confidence interval (CI), 57.6-62.6%), 79.2% (95% CI, 76.6-81.5%), and 0.56 at the LN level, respectively. At the patient level, the model achieved a sensitivity, specificity, and PPV of 81.1% (95% CI, 76.5-85.0%), 75.6% (95% CI, 65.1-83.8%), and 93.2% (95% CI, 89.7-95.6%), respectively. The model achieved a strong correlation and good consistency between the short diameter and volume of the automatically segmented and manually annotated LNs.

**Conclusion:**

This 3D V-Net model can segment LNs effectively based on pelvic DWI images for PCa and holds great potential for facilitating N-staging in clinical practice.

**Supplementary Information:**

The online version contains supplementary material available at 10.1186/s40644-025-00840-w.

## Introduction

Pelvic lymph nodes (LNs) are the most common location for prostate cancer (PCa) dissemination. LN invasion was confirmed in up to 15% of patients undergoing pelvic LN dissection (PLND) [1]. It is critical for clinical decision-making to accurately identify the number and location of LNs and assess the nodal metastatic burden before treatment. Negative pretreatment reporting would mean that surgery or radiation therapy may be limited to the prostate and not necessary for PLND, while positive pretreatment reporting would indicate the presence of other options, such as extended radiation therapy, PLND, or androgen deprivation therapy [1, 2, 3, 4].

However, the ideal imaging method does not yet exist. Although some functional MR imaging and targeted PET/CT imaging improve the N-staging of PCa, they are not currently a substitute for PLND [5]. MpMRI has been recognized as the first choice for PCa screening, local staging, and image-guided biopsy. DWI images with high b-values have strong diffusion effects and can suppress the signal of the background tissue. Thus, the pelvic LNs can be displayed and easily identified. Reporting N staging is a routine task in interpreting prostate mpMRI. Based on traditional size and shape assessment, mpMRI can detect LN metastasis with high specificity but low and heterogeneous sensitivity in the range of 40-60% [6]. Radiologists often use a short diameter of 8 mm as the threshold for suspected metastatic LNs and highlight them in the MR report [7], but false positive (FP) results can result when LNs are enlarged from conditions other than metastasis (such as hyperplasia). In addition, even LNs with a short diameter of less than 8 mm may harbor microscopic metastasis [8, 9], suggesting that small LNs should not be ignored. Thus, the identification of all LNs in the scan area is a preliminary step for further analysis of metastasis.

Identifying all pelvic LNs, especially tiny LNs, from numerous medical images is a time-consuming and experience-dependent process in a radiologist’s daily workflow. Therefore, there is a growing need for automatic pelvic LN identification. Convolutional neural networks (CNNs) have emerged as promising tools for automatic diagnosis and quantitative evaluation based on deep learning methods. V-Net [10], a fully CNN originally developed for prostate segmentation, has been successfully applied to various medical image segmentation tasks due to its stable and robust performance [11, 12]. However, the efficiency of applying the V-Net framework for LN segmentation is still unknown.

In this work, we attempt to develop an automated LN segmentation model on pelvic DWI images using the V-Net framework, and then we validate it on external datasets from multiple vendors and multiple centers.

## Materials and methods

The retrospective study herein was approved by Committee for Medical Ethics, Peking University First Hospital, with the requirement for written informed consent waived. The study protocol was assigned number 2021(060).

### Study subjects

A dataset of patients suspected of having PCa between February 2014 and March 2022 was obtained from Peking University First Hospital for model development. The inclusion criteria were as follows: (1) patients with biopsy-confirmed PCa or biopsy-negative patients who did not show underlying PCa within one year of clinical follow-up; (2) patients whose high b-value (≥ 800 s/mm^2^) DWI images were available; and (3) patients without a history of surgery, radiation, or adjuvant therapy for PCa before mpMRI. DWI images with ineligible quality were excluded. A total of 1151 patients with 1309 high b-value DWI images were finally recruited for model development.

**Fig. 1:**
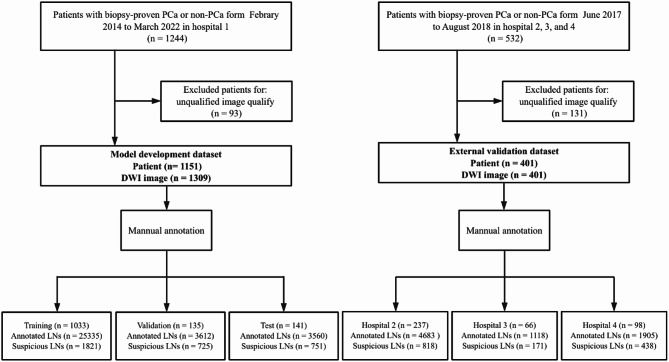
Flowchart of patient enrollment. Hospital1 refers to Peking University First Hospital. Hospital2 refers to the Second Affiliated Hospital of Dalian Medical University. Hospital3 refers to Fujian Medical University Union Hospital. Hospital4 refers to Jiaxing Hospital

An external dataset was included from three other hospitals (Second Affiliated Hospital of Dalian Medical University, Fujian Medical University Union Hospital, and Jiaxing Hospital) between June 2017 and August 2018 following the same inclusion and exclusion criteria. A total of 401 patients with 401 high b-value DWI images were finally enrolled for external validation. Figure 1 shows the flowchart of patient enrollment. All data were deidentified before enrollment, and clinical information, such as age and PSA level, was recorded for each enrolled patient.

### MRI protocol

The mpMRI data used for model development were obtained with seven 3.0-Tesla scanners and two 1.5-Tesla scanners. The mpMRI data used for external validation were obtained using six 3.0 Tesla scanners and three 1.5 Tesla scanners. Details of the DWI image protocols at each hospital are summarized in Supplementary Material Table S1.

### Annotations and reference standard

The format of DWI images was converted from DICOM to NIFTI. The annotation platform is the open-source software ITK-SNAP (version 3.6 2015; available at www.itksnap.org). All discernible LNs were annotated by the junior radiologist (5 years of experience) as Mask (1) The expert urogenital radiologist (35 years of experience) subsequently modified the annotations as Mask (2) Mask 2 was regarded as the reference standard for model development and external segmentation assessment. LNs with a short diameter > 8 mm are considered suspicious for metastasis, and the annotations of these LNs are used as the reference standard for assessing LN level detection. If a patient contains at least one LN suspicious for metastasis, the patient is considered a suspicious patient (known as N1) and is used as the reference standard for assessing patient-level detection.

### Preprocessing

B-spline interpolation to the third order was employed for all MR image interpolation tasks. All input images were cropped to 32 × 256 × 256 (z, y, x). The region of interest was then normalized into the range of [0, 1]. Histogram equalization is used to enhance image contrast.

### Data augmentation

Skewing (angel: 0–5), rotating (angel: 0–10), shearing (angel: 0–5), translation (scale: -0.1, 0.1), and adding noise to the images were exploited for data augmentation.

### Training model

**Fig. 2:**
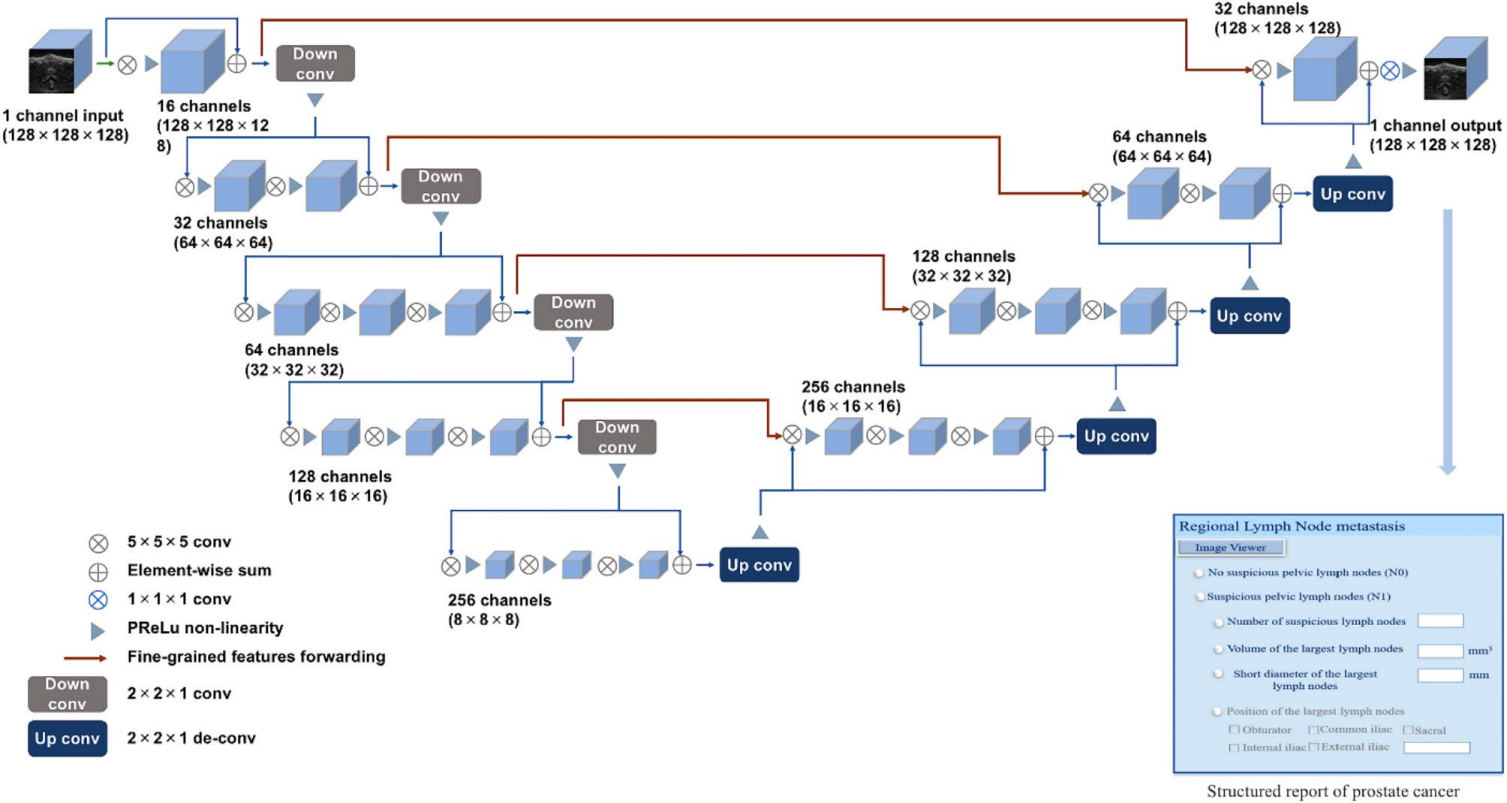
Model architecture based on 3D V-Net and result output

The 3D V-Net [10] serves as the foundational architecture for pelvic LN segmentation on DWI images (Fig. 2). This model was an application of a pre-existing framework originally proposed for prostate segmentation tasks. Inspired by the U-Net architecture [13] and the capabilities of fully convolutional neural networks, this network is tailored for processing MRI volumes with end-to-end training. V-Net, which serves as the baseline model, features four levels comprising encoding and decoding paths, as well as skip connections that operate within and across the paths. Unlike conventional methods that process input volumes slice by slice, the 3D V-Net employs volumetric convolutions for enhanced accuracy. The 1309 DWI images were randomly divided into the training set (*n* = 1033), validation set (*n* = 135), and testing set (*n* = 141). The network was trained with DWI images and their corresponding manual annotations on an Ubuntu 16.04 computer with GPU NVIDIA Tesla P100 16G, with 32 GB available in RAM. The software and packages used included Python 3.6, Opencv 3.4.0.12, Pytorch 0.4.1, SimpleITK 1.2.0, and Numpy 1.16.2. Using the Adam optimizer, the training of layers was conducted by stochastic gradient descent in a fixed batch size of three images. The learning rate was set as 0.0001. The network was trained for 400 epochs until the validation loss function was no longer decreasing.

### LN measurement and radiology report production

The contiguous voxel cluster predicted by this model was defined as an independent LN. Based on the segmentation results, the volume and short diameter of each segmented LN were automatically calculated by summing the pixel volumes and using the minimum-volume bounding box algorithm, respectively. Next, a structured radiology report (Fig. 2) was automatically filled in containing information on the number of LNs with a short diameter exceeding 8 mm (suspicious LNs), along with the short diameter and volume of the largest LN. When the model detected at least one suspicious LN, the radiology report was automatically filled as N1. Alternatively, if no suspicious LN was detected, the N-staging was automatically filled as N0.

### Evaluation criteria for LN segmentation

Model segmentation results were quantitatively compared with manual segmentation using the Dice similarity coefficient (DCS). For a further quantitative estimation of the 3D V-Net segmentation effectiveness, we calculated and compared the mean short diameter and volume of LNs in the reference standard and automatic segmentation. The segmentation performance of the model was evaluated in both internal test and external validation datasets at the levels of all LNs, suspicious LNs, and largest LNs.

### Evaluation criteria for LN detection

According to the guidelines, suspicious LNs with a short diameter greater than 8 mm must be reported [7]. A detection approach for suspicious LNs and suspicious patients was defined based on automatic segmentation [14]. We assessed the performance of the model in detecting suspicious LNs at both the LN and patient levels. At the LN level, we calculated the sensitivity, positive predictive value (PPV), and per-patient false-positive rate (FP/vol) to evaluate the model’s ability to detect suspicious LNs. At the patient level, we calculated the sensitivity, specificity, and PPV to evaluate the model’s ability to correctly identify patients with the N1 stage.

### Statistical analysis

Statistical analysis was performed using GraphPad Prism 8 (GraphPad Prism Software Inc., San Diego, CA) and SPSS (version 24.0, IBM Corp., Armonk, NY, USA). Normalized variables are presented as the mean ± standard deviation, and nonnormalized variables are presented as the median [Q1, Q3]. Categorical variables are presented as numbers (percentages). We used a one-way analysis of variance to compare the segmentation performance of the algorithm, i.e., DSC, and patient characteristics (age, tPSA level, LN volume, and short diameter). Post hoc multiple comparisons were conducted using the least significant difference. We conducted Wilcoxon signed-rank, Pearson correlation, and Bland‒Altman analyses to compare manual and automated segmentation of the short diameter and volume of LNs. All statistical tests were two-tailed with a 5% level of significance.

## Results

### Patient characteristics

**Fig. 3:**
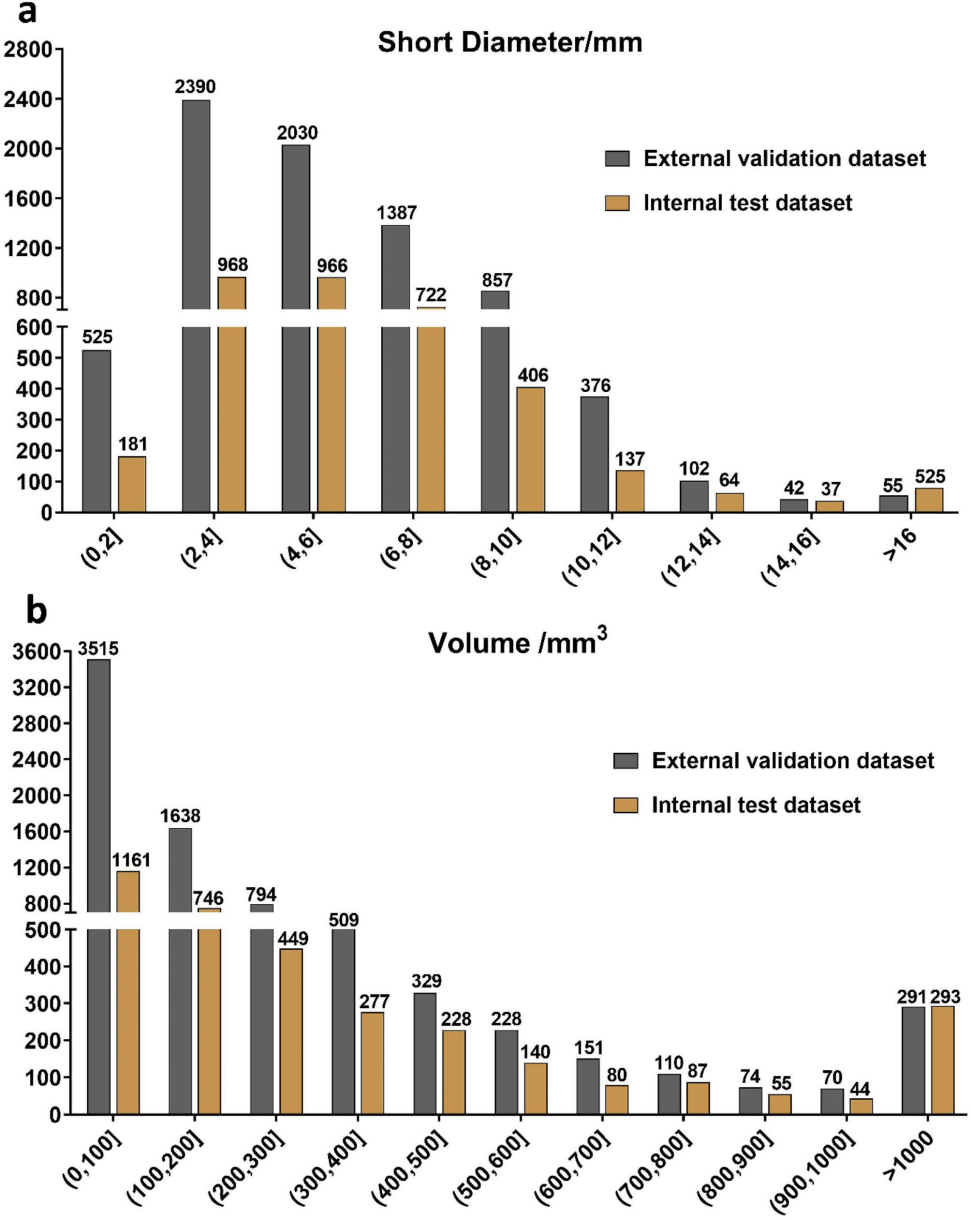
The distribution of lymph node short diameters (**a**) and volumes (**b**)

Table 1 displays the characteristics of the included patients. In the model development dataset, we annotated a total of 32,887 visible LNs, with 25,659 in the training set (24.8 per patient on average), 3,632 in the validation set (25.8 per patient on average), and 3,596 in the testing set (26.6 per patient on average). Additionally, we annotated 7,707 visible LNs in the external validation dataset, consisting of 401 patients, including 282 PCa patients and 119 non-PCa patients. Figure 3 presents the results of the statistical analysis of the short diameter and volume of the LNs in both the internal test dataset and external validation dataset.

**Table 1:** Clinical characteristics of the patients

Parameter	Model development dataset					External validation dataset					*P*
	Training	Validation	Test	Overall	*P*	Hospital 2	Hospital 3	Hospital 4	Overall	*P*	
**No. of patients**	919 (79.8)	116 (10.1)	116 (10.1)	1151	-	237 (59.1)	66 (16.5)	98 (24.4)	401	-	-
**No. of PCa patients**	820 (79.5)	104 (10.1)	107 (10.4)	1031	-	164 (58.2)	52 (18.4)	66 (23.4)	282	-	-
**No. of non-PCa patients**	99 (82.5)	12 (10.0)	9 (7.5)	120	-	73 (61.3)	14 (11.8)	32 (26.9)	119	-	-
**No. of DWI images**	1033 (78.9)	135 (10.3)	141 (10.8)	1309	-	237 (59.1)	66 (16.5)	98 (24.4)	401	-	-
**Age (years)**	70.0 ± 8.3	68.5 ± 9.6	69.1 ± 8.5	69.7 ± 8.5	0.971	71.33 ± 7.4	70.7 ± 7.2	72.1 ± 8.5	71.4 ± 7.6	0.394	0.000
**tPSA (ng/ml)**	16.7 [9.0, 56.4]	16.5 [8.4, 44.7]	11.3 [7.4, 43.2]	16.1 [8.8, 51.2]	0.262	19.1 [9.8, 63.9]	21.6 [8.4, 100.0]	16.9 [8.5, 61.4]	18.9 [9.3, 67.5]	0.734	0.161
**No. of annotated LNs**	25,335	3612	3560	32,507	-	4683	1118	1905	7707	-	-
**No. of suspicious LNs**	4821	725	751	6297	-	818	171	438	1427	-	-
**Average LNs per patient**	24.8 ± 9.3	25.8 ± 8.6	26.6 ± 9.3	25.1 ± 9.3	0.075	19.9 ± 8.2	16.7 ± 8.2	20.5 ± 8.0	19.5 ± 8.2	0.019	0.000
**Short diameter of largest LNs (cm)**	7.8 [6.0, 9.5]	8.7 [6.6, 10.3]	8.2 [6.4, 10.2]	7.9 [6.1, 9.7]	0.002	9.9 [8.4, 11.4]	9.9 [7.2, 13.2]	10.6 [9.2, 13.3]	10.0 [8.4, 12.1]	0.003	0.000
**Volume of largest LNs (cm** ^**3**^ **)**	5.8 [3.7, 11.3]	6.5 [4.0, 13.1]	6.0 [4.2, 15.2]	5.9 [3.8, 11.5]	0.064	0.8 [0.5, 1.3]	0.8 [0.5, 1.0]	0.7 [0.4, 1.2]	0.8 [0.5, 1.3]	0.120	0.000

tPSA = total prostate-specific antigen, LN = lymph node

The categorical variables are given as numbers (percentages). Quantitative variables were given as the median [Q1, Q3] for nonnormalized data

### Segmentation performance of the model

**Fig. 4:**
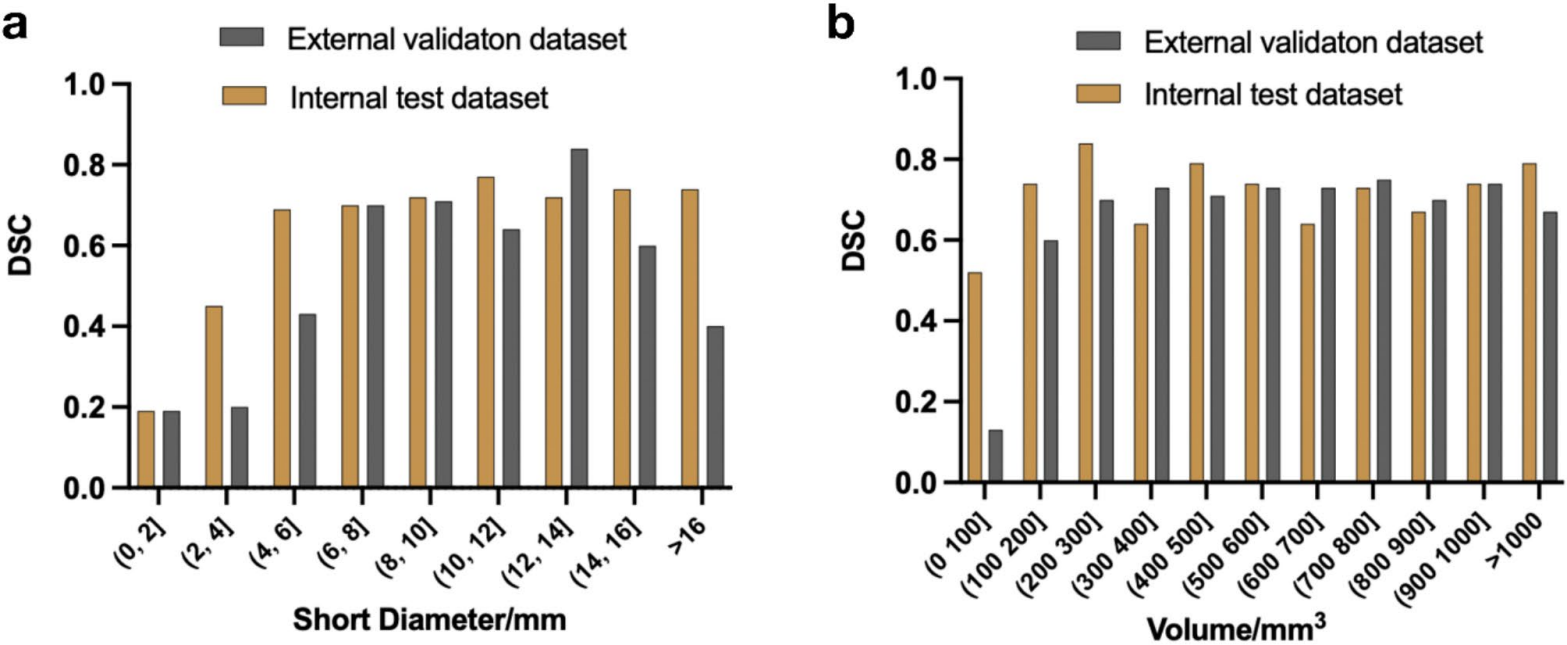
Dice similarity coefficient distribution of lymph nodes with different short diameters (**a**) and volumes (**b**) in internal and external validation datasets. DSC Dice similarity coefficient

We evaluated the segmentation results of the LNs on both the internal test dataset and the external validation dataset. Table 2 shows that the DSC values of all LNs were significantly lower than those of the suspicious and largest LNs in both datasets (all with *P* < 0.05). The largest LNs had the highest DSC value of 0.90 [0.75, 0.93] in the internal test dataset, indicating optimal segmentation performance. In contrast, there was no significant difference in the DSC values between the suspicious and largest LNs in the external validation dataset (0.82 [0.59, 0.92] vs. 0.82 [0.49, 0.92], *P* = 0.330). Figure 4 illustrates the distribution of DSC values among LNs with different short diameters and volumes in both the internal test dataset and external validation dataset.

**Table 2:** Segmentation result of the model

DSC	All LNs	Suspicious LNs	Largest LNs	*P* value		
				All vs. Suspicious	All vs. Largest	Suspicious vs. Largest
Internal test dataset	0.78 [0.51, 0.93]	0.82 [0.63, 0.91]	0.90 [0.75, 0.93]	< 0.001	< 0.001	0.015
External validation dataset	0.77 [0.37, 0.90]	0.82 [0.59, 0.92]	0.82 [0.49, 0.92]	< 0.001	< 0.001	0. 330

Suspicious LNs indicates the LNs larger than 0.8 cm in the shortest diameter

LNs lymph nodes

Quantitative variables were given as the median [Q1, Q3] for nonnormalized data

### Quantitative evaluation of segmentation performance

**Fig. 5:**
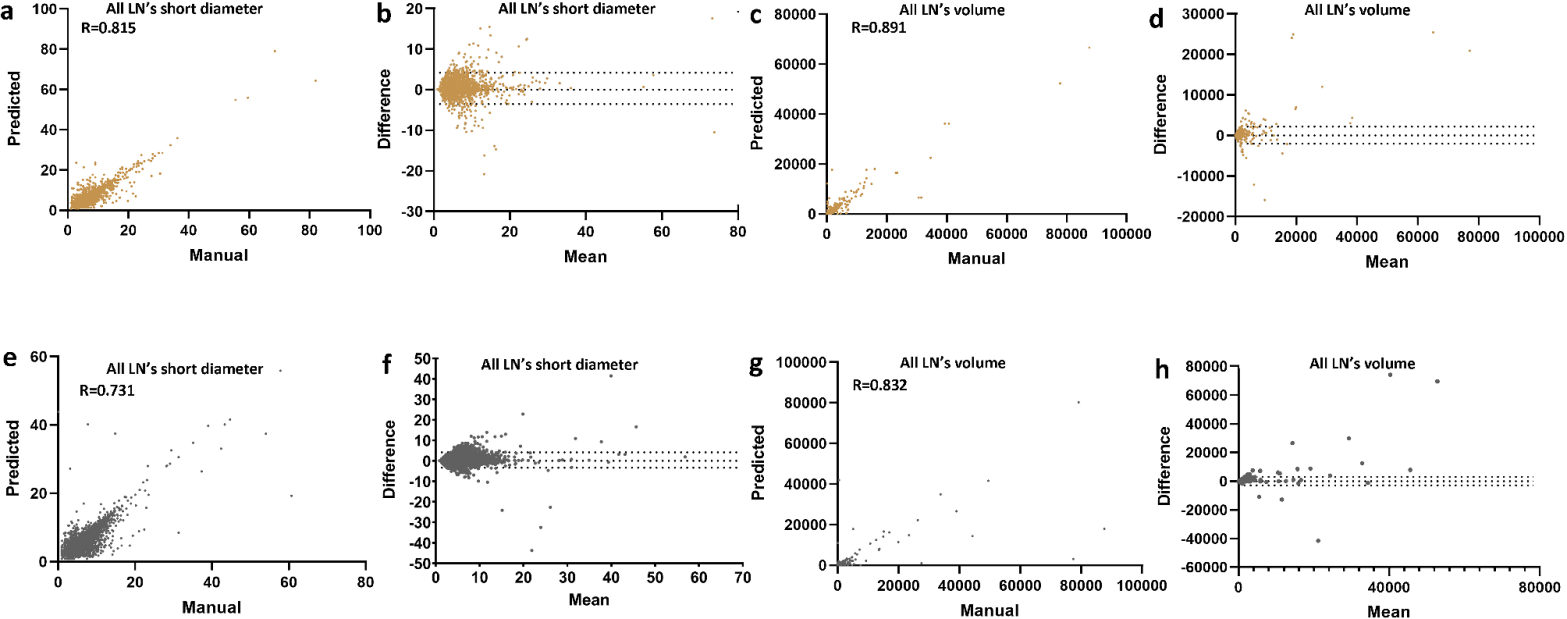
Quantitative comparison of the short diameter and volume of the lymph nodes. Correlation and Bland–Altman plots of lymph node short diameter and volume between automated segmentation and manual segmentation for the internal test dataset (**a**–**d**) and external validation dataset (**e**–**h**). LN lymph node

Table 3 summarizes the median short diameter and volume measurements for all LNs, suspicious LNs, and the largest LNs. Figure 5 presents a quantitative comparison of the LNs’ short diameter and volume between automated and manual segmentation. We found a strong correlation between the short diameter (*R* = 0.731–0.815) and volume (*R* = 0.832–0.891) of the automatically segmented LNs and the manually annotated LNs. Our Bland–Altman analysis showed good consistency between the automated segmentation and manual annotation of all LNs, suspicious LNs, and largest LNs, with most values falling within the consistency interval.

**Table 3:** Quantitative measurements between automated segmentation and manual annotation

Quantitative metrics	All LNs			Suspicious LNs			Largest LNs		
	Automated segmentation	Manual annotation	*P*	Automated segmentation	Manual annotation	*P* value	Automated segmentation	Manual annotation	*P* value
Internal test dataset									
Volume (mm^3^)	172.3 [71.1, 390.3]	197.9 [83.0, 449.2]	0.002	623.0 [374.3, 1123.1]	785.1 [517.9, 1389.3]	0.000	10.3 [8.7, 13.8]	10.8 [9.4, 15.1]	0.000
Short diameter (mm)	5.4 [3.9,7.3]	5.7 [4.0,7.7]	0.000	9.1 [7.7, 11.2]	9.7 [8.7, 11.9]	0.000	752.3 [466.4, 1548.5]	886.2 [530.8, 1787.2]	0.000
External validation dataset									
Volume (mm^3^)	103.3 [45.9, 244.90]	130.3 [61.2, 304.2]	0.000	428.6 [244.9, 688.8]	551.0 [382.7, 870.9]	0.000	212.2 [95.1, 382.6]	266.0 [134.7, 463.2]	0.000
Short diameter (mm)	4.8 [3.4, 6.9]	5.1 [3.6, 7.5]	0.000	8.7 [7.4, 10.1]	9.4 [8.6, 10.7]	0.000	6.3 [4.7, 8.1]	6.8 [5.0, 8.6]	0.000

LNs lymph nodes

### LN detection based on segmentation

**Fig. 6:**
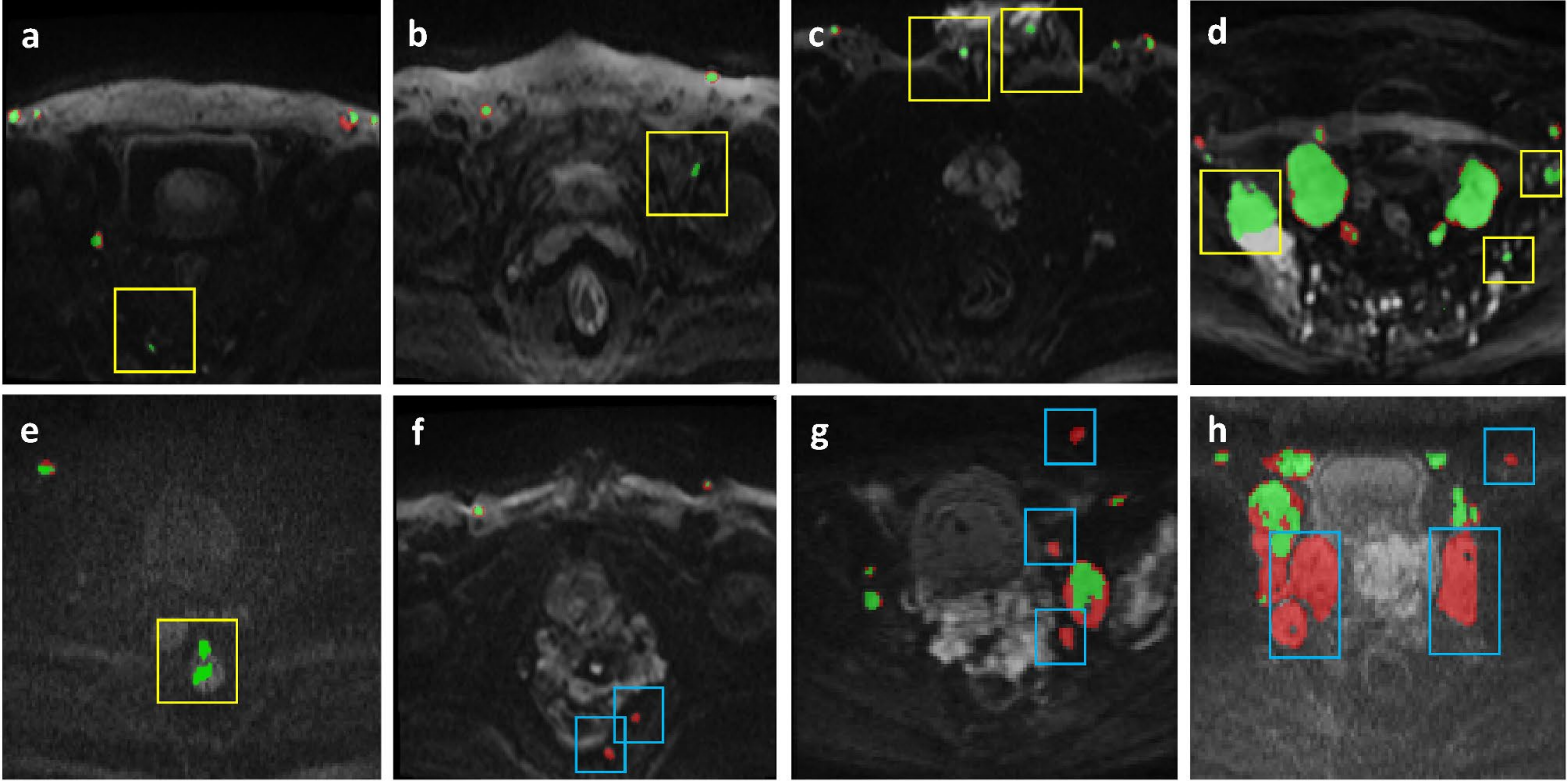
Examples of the segmentation results of the model based on 3D V-Net for the lymph nodes. The reference standard of the manual annotation is represented by the red area, while the predicted region of the model is indicated in green. The area of overlap is shown in green as well. False positive segmentation results are circled in yellow boxes (**a-e**), while false negative segmentation results are circled in blue boxes (**f-h**)

Table 4 presents the detection results for suspicious LNs and patients (based on the largest LNs) in both the internal test and external validation datasets. In the internal test dataset, our model demonstrated good performance in detecting suspicious LNs, achieving a positive predictive value (PPV) of 79.8% (95% confidence interval (CI), 76.5-82.8%), a sensitivity of 66.6% (95% CI, 63.1-69.9%), and a false positive/volume (FP/vol) of 1.07. Our model also achieved good performance in detecting suspicious patients, with a PPV of 98.0% (95% CI, 92.9-99.4%), sensitivity of 89.0% (95% CI, 81.7-93.6%), and specificity of 71.4% (95% CI, 35.9-91.8%). In the external validation dataset, our model also demonstrated good performance in detecting suspicious LNs, with a PPV of 79.2% (95% CI, 76.6-81.5%), sensitivity of 60.1% (95% CI, 57.6-62.6%), and a lower false positive rate of 0.56. Additionally, our model achieved good performance in detecting suspicious patients, with a PPV of 93.2% (95% CI, 89.7-95.6%), sensitivity of 81.1% (95% CI, 76.5-85.0%), and specificity of 75.6% (95% CI, 65.1-83.8%). Figure 6 shows examples of the LN detection results obtained with the model. FPs typically occur due to high-intensity structures such as nerve tissue (Fig. 6a), hip joint (Fig. 6b), spermatic cord (Fig. 6c), and bone metastasis (Fig. 6d), as well as rectum lesions (Fig. 6e), among others. False negative (FN) predictions may result from the misattribution of small lesions or insufficient contrast compared to the background. In cases of diffuse PCa, perirectal and periprostatic LNs are commonly missed, resulting in FNs (Fig. 6f and g). Additionally, obvious swelling and necrosis of LNs can also be easily overlooked (Fig. 6h).

**Table 4:** Detection results of suspicious lymph nodes and patients

	Internal test dataset		External validation dataset	
	Suscipious LNs	Suscipious Patients	Suscipious LNs	Suscipious Patients
No. of TP	491	97	858	262
No. of FP	124	2	226	19
No. of FN	246	12	569	61
No. of TN	NA	5	NA	59
PPV (95%CI)	79.8% (76.5%, 82.8%)	98.0% (92.9%, 99.4%)	79.2% (76.6%, 81.5%)	93.2% (89.7%, 95.6%)
Sensitivity (95% CI)	66.6% (63.1%, 69.9%)	89.0% (81.7%, 93.6%)	60.1% (57.6%, 62.6%)	81.1% (76.5%, 85.0%)
Specificity (95% CI)	NA	71.4% (35.9%, 91.8%)	NA	75.6% (65.1%, 83.8%)
FP/vol	1.07	-	0.56	-

LNs lymph nodes, TP true positive, FP false positive, FN false negative, PPV positive predictive value, CI Confidence Interval, FP/vol per-patient false-positive rate

## Discussion

N-staging is a critical factor in determining treatment options and predicting patient outcomes. The initial step in this process is identifying all LNs, which can be a tedious and time-consuming task. Our study introduces a deep learning model that enables the accurate detection and segmentation of all pelvis LNs on DWI images. Furthermore, we validated the model’s performance on an external dataset.

A comprehensive evaluation of size, morphological features, signal intensity, and other imaging parameters is essential in the interpretation of pelvis LNs in the context of PCa N staging. The PIRADS guidelines [7] recommend reporting a short diameter greater than 8 mm as a threshold for suspected metastatic LNs. This approach oversimplifies the complex nature of LN metastasis. Of note, LNs with short diameters greater than 8 mm can exhibit benign characteristics, while those with short diameters less than 8 mm may still harbor metastatic cells [8, 9]. Our study developed a model capable of segmenting all visible LNs on DWI images, whether they are healthy or metastatic. Furthermore, we exploited a cutoff threshold for LNs with a short diameter of more than 8 mm, allowing us to assess the performance of our model in detecting suspicious metastatic LNs. In the external validation test dataset, the model achieved a DSC of 0.77 for all LNs and 0.82 for suspicious LNs. The model achieved a sensitivity of 60.1%, PPV of 79.2%, and FP/vol of 0.56 for detecting suspicious LNs at the LN level. The results from our external validation dataset confirmed the feasibility of this method, which could aid in LN staging, quantitative measurements of tumor burden, and image-guided treatment of patients with PCa.

In clinical practice, radiologists commonly focus on measuring and recording the short diameter and volume of the largest LN as it correlates with the N stage of the patient. Therefore, we took this factor into consideration in our study to ensure its practicality. We assessed the model’s ability to detect and segment the largest LNs to enhance the clinical relevance of our analysis. In the external validation test dataset, the model demonstrated a DSC of 0.82 for the largest LNs. At the patient level, the model exhibited a sensitivity of 81.1%, specificity of 75.6%, and positive predictive value (PPV) of 93.2% in detecting patients with suspicious LNs. Furthermore, we leveraged quantitative measurements of the largest LN’s short diameter and volume to automatically generate N-staging, which was then automatically included in the structured report on PCa.

Among neural network structures, fully convolutional networks (FCNs) [15], U-Net [13], 3D U-Ne t [16], and V-Net [10] are the most widely used architectures. The FCN [15], which adopts an end-to-end convolutional neural network and deconvolution for up-sampling, was the first to pioneer image segmentation and deep learning techniques. However, its low sensitivity to image details and tendency to cause partial information loss result in low segmentation accuracy for small structures. Ronneberger et al. proposed the U-Net [13] method, based on FCN [15], which applies a fully convolutional network to medical image segmentation. Unfortunately, FCN [15] and U-Net [13] can only be used for the identification and segmentation of two-dimensional images, whereas 3D U-Net [13] and V-Net [10] can process three-dimensional images. Of the two, V-Net [10] training has become the primary method of medical image segmentation due to its high speed and short completion time. In this study, even with significant individual variation in size, pose, shape, and sparsely distributed location of pelvic LNs, we demonstrate that V-Net’s outstanding performance can be extended to the challenging task of LN segmentation by utilizing an ensemble strategy.

Liu et al. [14] developed a 3D U-Net model that can detect and segment all pelvic LNs on DWI images. The model achieved a high recall value of 0.98 for identifying suspicious LNs. However, their research data are limited and lack external validation. In a similar vein, Zhao et al. [17] presented an innovative autoLNDS model to detect and segment LNs with a short diameter greater than 3 mm on MR examination (T2-weighted imaging and DWI). Their external testing showed that the model achieved a sensitivity, PPV, and FP/vol of 62.6%, 64.5%, and 8.2, respectively, which is comparable to our results. However, their dataset size (293 patients) was smaller than the natural detection task dataset. Their training and internal testing datasets were generated by the same MR vendor from one medical center, which limits the variability of the dataset. In contrast, our model development dataset was generated by eight MR scanners from a single hospital, including 1,151 patients, while the external validation dataset included 401 patients generated by seven scanners from four hospitals. This dataset is large and heterogeneous compared to other studies of its kind, which enhances the robustness and generalizability of our model.

Radiomics technology holds promise in predicting pelvic LN metastasis in various malignancies, including PCa [18, 19, 20, 21]. Radiomics-based pelvic LN metastasis prediction models typically undergo a multistep process, including segmentation of the region of interest (ROI), extraction of quantitative features, feature selection, and model building. Within the field, researchers have multiple choices when selecting an ROI to study, including the prostate glands, PCa foci, or LNs. Among these options, LNs emerge as the most frequently investigated ROI. A fundamental premise of these studies is to initially segment all LNs, and our study represents an initial step towards this goal, providing an automated method for delineating the ROI of LNs, thus addressing the current limitation of relying on manual delineation at this stage.

While the model achieved acceptable accuracy for the detection of suspicious metastases patients, further improvements are needed to increase its sensitivity at the individual LN level. False positives and false negatives are still common. Lymphadenopathies in the pelvis exhibit great heterogeneity in terms of shape and size, which makes it difficult to accurately distinguish true LN regions from other regions. Furthermore, the relatively small size of LN lesions in comparison to the background volume creates an imbalance that further complicates segmentation. This imbalance also results in a large number of FPs with no specificity for high-intensity mimics, which ultimately lowers the overall specificity of the segmentation process. While larger LNs tend to produce better segmentation results [17], there is a risk of FN detection due to obvious swelling and necrosis. This can be especially problematic in cases of diffuse PCa that occupy most of the pelvic cavity. To address the issue of imbalanced data, we utilized the Dice coefficient as the loss function in the 3D V-Net al.gorithm. We also manually annotated all visible LNs to capture as many specific voxel details as possible. In analyzing the results, we discovered instances where the model made accurate predictions, despite the reference standard failing to annotate them. In the annotation process of the reference standard, the junior radiologist provided a fresh perspective and attention to detail, while the expert radiologist provided valuable insights and corrections. Despite the limitations of manual annotation, which can vary both within and between operations, it remains the most reliable method for accurate image segmentation, and there is currently no viable substitute. Our findings suggest that V-Net can be an effective tool for LN segmentation despite the challenges posed by the complex nature of these lesions.

Several limitations of our study should be acknowledged. First, our study lacked one-to-one MR-surgical pathological LN confirmation. This challenge arises due to the selective use of PLND in clinical practice, particularly for patients with low-risk PCa or metastatic disease where PLND may not be routinely recommended. This does not diminish the validity of our current findings. Future studies may benefit from incorporating histopathologically confirmed metastatic lymph nodes for further analysis of model performance. Second, while our reference standards were established by a senior radiologist, inviting reputable senior radiologists from well-known clinical centers could enhance the credibility of our study by establishing a more robust ground reference. Third, we focused on the feasibility of multi-device image segmentation of pelvic LNs. However, there was a failure to address the relative intensity problem with MRI and to perform any corrections aimed at minimizing discrepancies between different scanners at different magnets. Incorporating these measures in future studies may enhance the reliability of the model results.

## Conclusion

In conclusion, we developed a 3D V-Net model and evaluated its performance on both internal and external validation datasets, demonstrating its feasibility for automated detection and segmentation of pelvic LNs on DWI images. This presents a promising step toward a clinically useful deep learning-based tool that can provide an objective and comprehensive assessment of tumor burden in patients with PCa.

## Electronic supplementary material

Below is the link to the electronic supplementary material.

Supplementary Material 1

## Data Availability

The datasets used and/or analyzed during the current study are available from the corresponding author on reasonable request.

## Abbreviations

LN: Lymph node
PCa: Prostate cancer
DWI: Diffusion-weighted imaging
DSC: Dice similarity coefficient
PPV: Positive predictive value
FP/vol: Per-patient false-positive rate
CI: Confidence interval
PLND: Pelvic lymph node dissection
FP: False positive
CNN: Convolutional neural network
FN: False negative
FCN: Fully convolutional network
ROI: region of interest
