# Supplementary material for: Deep learning-based fully automated detection and segmentation of pelvic lymph nodes on diffusion-weighted images for prostate cancer: a multicenter study
Source: Cancer Imaging. 2025 Mar 17;25:37. doi: 10.1186/s40644-025-00840-w (PMC11912796; doi:10.1186/s40644-025-00840-w)
Supplement: Supplementary file 1 — Supplementary Material 1 [file 40644_2025_840_MOESM1_ESM.docx]

**Supplementary Material**

**Table S1 Imaging protocols of the pelvis DWI sequences**

| **Parameter** | **Model development dataset** | | | |  | **External validation dataset** | | | |
| --- | --- | --- | --- | --- | --- | --- | --- | --- | --- |
|  | **Training**  **(N=1033)** | **Validation**  **(N=141)** | **Test**  **(N=135)** | **Overall**  **(N=1309)** |  | **Hospital 2**  **(N=237)** | **Hospital 3**  **(N=66)** | **Hospital 4**  **(N=98)** | **Overall**  **(N=401)** |
| **Model Name** |  |  |  |  |  |  |  |  |  |
| Achieva | 41 (4.0) | 8 (5.7) | 5 (3.7) | 54 (4.1) |  | 0 (0.0) | 0 (0.0) | 0 (0.0) | 0 (0.0) |
| Aera | 92 (8.9) | 12 (8.5) | 8 (5.9) | 112 (8.6) |  | 0 (0.0) | 0 (0.0) | 0 (0.0) | 0 (0.0) |
| DISCOVERY MR750 | 695 (67.3) | 93 (66.0) | 92 (68.1) | 880 (67.2) |  | 0 (0.0) | 0 (0.0) | 0 (0.0) | 0 (0.0) |
| DISCOVERY MR750w | 0 (0.0) | 0 (0.0) | 0 (0.0) | 0 (0.0) |  | 82 (34.6) | 5(7.6) | 0 (0.0) | 87 (21.7) |
| lngenia | 66 (6.4) | 12 (8.5) | 19 (14.1) | 97 (7.4) |  | 0 (0.0) | 0 (0.0) | 0 (0.0) | 0 (0.0) |
| lngenia CX | 0 (0.0) | 0 (0.0) | 0 (0.0) | 0 (0.0) |  | 0 (0.0) | 2 (3.0) | 0 (0.0) | 2 (0.5) |
| MAGNETOM_ESSENZA | 0 (0.0) | 0 (0.0) | 0 (0.0) | 0 (0.0) |  | 0 (0.0) | 0 (0.0) | 98 (100.0) | 99 (24.7) |
| Multiva | 42 (4.1) | 1 (0.7) | 2 (1.5) | 45 (3.4) |  | 0 (0.0) | 0 (0.0) | 0 (0.0) | 0 (0.0) |
| Prisma | 1 (0.1) | 0 (0.0) | 0 (0.0) | 1 (0.1) |  | 0 (0.0) | 45 (68.2) | 0 (0.0) | 45 (11.2) |
| Signa HDxt | 47 (4.5) | 11 (7.8) | 6 (4.4) | 64 (4.9) |  | 2 (0.8) | 0 (0.0) | 0 (0.0) | 2 (0.5) |
| Skyra | 0 (0.0) | 0 (0.0) | 0 (0.0) | 0 (0.0) |  | 90 (38.0) | 0 (0.0) | 0 (0.0) | 90 (22.4) |
| TrioTim | 0 (0.0) | 0 (0.0) | 0 (0.0) | 0 (0.0) |  | 0 (0.0) | 13 (19.7) | 0 (0.0) | 13 (3.2) |
| uMR 790 | 49 (4.7) | 5 (3.5) | 2 (1.5) | 56 (4.3) |  | 0 (0.0) | 0 (0.0) | 0 (0.0) | 0 (0.0) |
| Verio | 0 (0.0) | 0 (0.0) | 0 (0.0) | 0 (0.0) |  | 63 (26.6) | 0 (0.0) | 0 (0.0) | 63 (15.7) |
| Amira | 0 (0.0) | 0 (0.0) | 0 (0.0) | 0 (0.0) |  | 0 (0.0) | 1 (1.5) | 0 (0.0) | 1 (0.2) |
| **Magnetic Field** |  |  |  |  |  |  |  |  |  |
| 1.5 T | 149 (14.1) | 18 (12.8) | 10 (7.4) | 177 (7.5) |  | 2 (2.3) | 1 (1.5) | 98 (100.0) | 101 (25.2) |
| 3 T | 884 (85.6) | 123 (87.2) | 125 (92.6) | 1132 (92.5) |  | 235 (97.7) | 65 (98.5) | 0 (0.0) | 300 (74.8) |
| **b Value, s/mm^2^** |  |  |  |  |  |  |  |  |  |
| 800 | 334 (32.3) | 55 (39.0) | 42 (31.1) | 431 (32.9) |  | 16 (6.8) | 4 (6.1) | 98 (100.0) | 118 (29.4) |
| 1000 | 16 (1.5) | 1 (0.7) | 4 (3.0) | 21 (1.6) |  | 92 (38.8) | 7 (10.6) | 0 (0.0) | 99 (24.7) |
| 1200 | 0 (0.0) | 0 (0.0) | 0 (0.0) | 0 (0.0) |  | 46 (19.4) | 0 (0.0) | 0 (0.0) | 46 (11.5) |
| 1400 | 678 (65.6) | 85 (60.3) | 89 (65.9) | 852 (65.1) |  | 14 (5.9) | 0 (0.0) | 0 (0.0) | 14 (3.5) |
| 1500 | 0 (0.0) | 0 (0.0) | 0 (0.0) | 0 (0.0) |  | 69 (29.1) | 18 (27.3) | 0 (0.0) | 87 (21.7) |
| 2000 | 5 (0.5) | 0 (0.0) | 0 (0.0) | 5 (0.4) |  | 0 (0.0) | 37 (56.1) | 0 (0.0) | 37 (9.2) |
| **Reconstruction Diameter, mm** | 517 [259, 775] | 240 [220, 360] | 240 [220, 360] | 240 [220, 360] |  | 221 [200, 240] | 250 [250, 250] | 240 [220, 240] | 240 [240, 320] |
| **Slice Thickness, mm** | 4.5 [4.0, 8] | 4.5 [4.0, 8.0] | 4.5 [4.0, 8.0] | 4.5 [4.0, 8.0] |  | 3.0 [3.0, 3.5] | 3.5 [3.5, 4.0] | 4.0 [4.0, 4.0] | 3.5 [3.0, 4.0] |
| **Slice Spacing, mm** | 4.5 [4, 9] | 4.5 [4.0, 9.0] | 4.5 [4.0, 9.0] | 4.5 [4.0, 9.0] |  | 3.3 [3.6, 4.2] | 3.5 [3.5, 4.8] | 4.8 [4.8, 4.8] | 4.0 [3.6, 4.8] |
| **Pixel Spacing, mm** | 1.0 [0.9, 1.4] | 1.4 [0.9, 0.4] | 1.0 [0.9,1.4] | 1.0 [0.9, 1.4] |  | 0.9 [0.9, 1.25] | 0.9 [0.9, 1.3] | 1.8 [1.8, 1.8] | 1.3 [0.9, 1.8] |
| **Matrix size -** **Rows** | 256 [256, 256] | 256 [256, 256] | 256 [256, 256] | 256 [256, 256] |  | 228 [136, 256] | 228 [200, 228] | 112 [112, 112] | 228 [112, 228] |
| **Matrix size - Columns** | 256 [256, 256] | 256 [256, 256] | 256 [256, 256] | 256 [256, 256] |  | 228 [160, 256] | 228 [192,228] | 112 [112, 112] | 228 [112, 228] |
| **TR, ms** | 3225 [2668, 4000] | 3500 [2675, 4000] | 3171 [2654, 4000] | 3243 [2668, 4000] |  | 4800 [4153, 5000] | 4900 [3400, 4900] | 3800 [3800, 3900] | 4500 [3800, 4900] |
| **TE, ms** | 59.9 [50.8, 61.4] | 55.4 [50.7, 61.4] | 60.3 [50.7, 61.7] | 59.7 [50.9, 61.1] |  | 73 [63, 77] | 62 [58, 69] | 81 [81, 81] | 75 [63, 81] |

TR Repetition time, TE Echo time

The categorical variables are given as numbers (percentages). Quantitative variables were given as the median [Q1, Q3] for nonnormalized data.
